# Bivalent oral cholera vaccine in participants aged 1 year and older in the Dominican Republic: A phase III, single-arm, safety and immunogenicity trial

**DOI:** 10.1080/21645515.2018.1430540

**Published:** 2018-02-22

**Authors:** Lina Cordero De Los Santos, Jesús Feris-Iglesias, Naveena Aloysia D'Cor, Venkata Jayanth Midde, Badri Narayan Patnaik, Yaël Thollot, Anvar Rasuli, Eric Desauziers

**Affiliations:** a CAIMED Dominicana S.A.S., Investigación en Salud, Santo Domingo, Dominican Republic; b Hospital Infantil Dr. Robert Reid Cabral, Centro de los Héroes, Departamento de Enfermedades Infecciosas, Santo Domingo, Dominican Republic; c Clinical R&D, Shantha Biotechnics Private Limited (A Sanofi Company), Basheerbagh, Hyderabad, Telangana, India; d Medical Department, Sanofi Pasteur, Lyon, France

**Keywords:** OCV, Shanchol™, bivalent oral cholera vaccine, Dominican Republic, cholera, safety, immunogenicity

## Abstract

The Dominican Republic, historically non-endemic for cholera, is experiencing an ongoing cholera epidemic. We assessed the safety and immunogenicity of two doses of the killed bivalent (O1 and O139) whole-cell oral cholera vaccine (OCV) on day (D)0 and D14 in healthy participants aged ≥1 year. Immediate unsolicited systemic adverse events (AEs) were monitored up to 30 minutes and solicited systemic reactions, up to 7 days after each vaccination. Unsolicited AEs were recorded up to D14 (post-dose 1) and 30 days post-dose 2. A vibriocidal antibody assay with microtiter technique was used to measure serum antibodies to *V. cholerae* strains (O1 El Tor Inaba, O1 El Tor Ogawa, O139) on D0, D14 and D28. Geometric mean titers (GMTs) and seroconversion (≥4-fold increase from D0) rates were calculated. We recruited 336 participants; 112 in three age groups (1–4, 5–14 and ≥15 years). No safety concerns were observed. GMTs increased from baseline for all serotypes, with marked increases for O1 Inaba and Ogawa post-dose 1. Post-dose 2 GMTs tended to be equal or slightly lower, with ranges: O1 Inaba, 283 (95% confidence interval 191–419) to 612 (426–880); O1 Ogawa, 346 (223–536) to 754 (553–1028); and O139, 20.3 (13.5–30.6) to 43.8 (30.1–63.7). Seroconversion rates post-dose 2 for O1 Inaba and Ogawa were high (≥87%) for all age groups. OCV demonstrated an acceptable safety profile and robust immunogenicity in these participants, in-line with previous observations in epidemic and endemic settings.This study is registered on www.clinicaltrials.gov (NCT02434822).

## Introduction

Cholera remains a threat to global public health, with 1.3–4.0 million cholera cases and 21,000–143,000 deaths estimated to have occurred annually between 2008 and 2012 in 69 cholera-endemic countries.^1^ Outbreaks occur in both endemic and epidemic settings, predominantly affecting developing countries across Africa, Asia and the Americas.^1^ Poor hygiene, sanitation and limited access to clean water in affected areas facilitate transmission of the water-borne pathogen *Vibrio cholerae*.

The killed bivalent (O1 and O139) whole-cell oral cholera vaccine (OCV) (Shanchol™, Shantha Biotechnics Pvt LTD, Hyderabad, India) was first licensed in India as a two-dose regimen in 2009. It is one of three OCVs that are now prequalified by the World Health Organisation (WHO) to help prevent or control cholera outbreaks.^2^ The safety and immunogenicity of OCV have been demonstrated mainly in the historically cholera-endemic countries of India and Bangladesh.^3^^,^^4^ Cumulative protective vaccine efficacy against cholera was demonstrated for up to 5 years following two doses of OCV in individuals aged over 1 year living in the urban slums of Kolkata, India, with an efficacy of 65% (95% confidence interval (CI) 52%–74%).^5^ OCV was also shown to be well tolerated and to elicit robust immune responses in Haiti and Ethiopia, which have recently been considered to be cholera-endemic countries.^1^^,^^6^^,^^7^ In Haiti, a further study demonstrated very high efficacy of OCV in reducing the number of culture-confirmed cases of cholera in an urban slum community from Port-au-Prince over a 37-month post-vaccination follow-up period, with an estimated vaccine efficacy of 97.5% compared with unvaccinated individuals from the same area.^8^ As part of the standard requirements for pre-qualified vaccines, the WHO has requested the assessment of the safety and immunogenicity of the OCV vaccine in Latin American countries in addition to Asia and Africa. The Dominican Republic, historically non-endemic for cholera, has been experiencing a cholera epidemic since identification of the first case in October 2010. A total of 33,160 cases of suspected cholera and 490 deaths were reported up to 2015.^9^ We assessed the safety profile and immunogenicity of two doses of OCV when administered 14 days apart to healthy participants aged 1 year or over in the Dominican Republic.

## Results

### Study population

**Figure 1.**
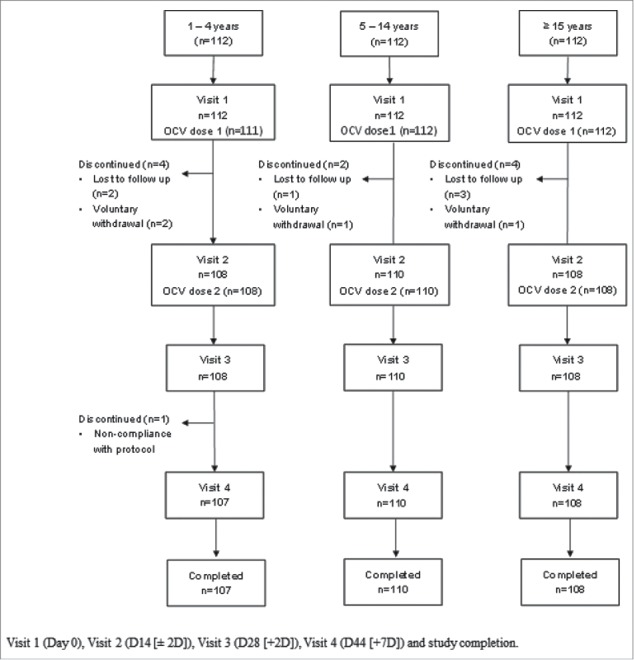
Participant disposition through the study.

A total of 336 healthy participants in the Dominican Republic were recruited, 112 in each age group (1–4 years, 5–14 years, and ≥15 years). All participants received at least one dose of OCV, the scheduled interval of 14 (+2 days) between vaccinations was reduced for 4 participants (all in the ≥15 years group) and exceeded for 10 participants (5 in the 1–4 years age group and 5 in the 5–14 age group). Participant baseline characteristics are summarized in Table 1. Eleven participants discontinued from the study early (5 from the 1–4 years, 2 from the 5–14 years, and 4 from the ≥15 years age group): one due to regurgitation of study vaccine; six were lost to follow-up; and four were voluntarily withdrawn, not due to an adverse event (AE) (Fig. 1).

**Table 1.** Participant baseline characteristics (Full Analysis Set).

	1–4 years	5–14 years	≥15 years	Overall
	(N = 112)	(N = 112)	(N = 112)	(N = 336)
Sex, n (%)				
Female	56 (50.0)	54 (48.2)	77 (68.8)	187 (55.7)
Age, years				
Mean (SD)	2.66 (1.05)	9.23 (2.87)	30.5 (9.50)	14.1 (13.2)
Racial origin, n (%)				
Mixed origin	112 (100)	112 (100)	112 (100)	336 (100)

### Safety and reactogenicity

Solicited systemic reactions were reported more frequently after the first dose than after the second dose (Table 2). The most commonly reported (i.e. ≥10% of participants in each age group) systemic reactions after either dose were: cough, fever and diarrhea in 1–4 year olds (17.6%, 14.8% and 10.2%, respectively); diarrhea in 5–14 year olds (10.9%); and abdominal pain, dryness of mouth and nausea in the ≥15 years age group (13.0%, 12.0% and 10.2%, respectively) (Table 2). The frequency of abdominal pain tended to increase with age, and there were more cases of rash in 1–4 year olds than the other age groups. Most solicited systemic reactions were of Grade 1 intensity and resolved within 3 days. There were no Grade 4 solicited systemic AEs reported. Grade 3 solicited systemic AEs were reported in 1.2% of participants post-dose 1 (2 in the 1–4; 1 in the 5–14; and 1 in the ≥15 years age group) and 2.8% of participants post-dose 2 (3 in the 1–4; 3 in the 5–14; and 3 in the ≥15 years age group). The Grade 3 solicited systemic reactions were mostly fever.

**Table 2.** Solicited systemic reactions within 7 days after each vaccination and unsolicited systemic AEs up to 30 days after dose 2 (Safety Analysis Set).

	1–4 years (N = 112)		5–14 years (N = 112)		≥15 years (N = 112)		All groups (N = 336)	
	n/M	% (95% CI)	n/M	% (95% CI)	n/M	% (95% CI)	n/M	% (95% CI)
Participants experiencing at least one								
Solicited systemic reaction								
Post-dose 1	28/107	26.2 (18.1–35.6)	27/110	24.5 (16.8–33.7)	30/108	27.8 (19.6–37.2)	85/325	26.2 (21.5–31.3)
Post-dose 2	25/108	23.1 (15.6–32.2)	13/110	11.8 (6.4–19.4)	16/106	15.1 (8.9–23.4)	54/324	16.7 (12.8–21.2)
Post-any dose	43	39.8 (30.5–49.7)	34	30.9 (22.4–40.4)	36	33.3 (24.6–43.1)	113	34.7 (29.5–40.1)
Fever	16	14.8 (8.7–22.9)	10	9.1 (4.4–16.1)	9	8.3 (3.9–15.2)	35	10.7 (7.6–14.6)
Nausea	6	5.6 (2.1–11.7)	5	4.5 (1.5–10.3)	11	10.2 (5.2–17.5)	22	6.7 (4.3–10.0)
Vomiting	7	6.5 (2.6–12.9)	4	3.6 (1.0– 9.0)	3	2.8 (0.6–7.9)	14	4.3 (2.4–7.1)
Diarrhea	11	10.2 (5.2–17.5)	12	10.9 (5.8–18.3)	10	9.3 (4.5–16.4)	33	10.1 (7.1–13.9)
Abdominal pain	6	5.6 (2.1–11.7)	10	9.1 (4.4–16.1)	14	13.0 (7.3–20.8)	30	9.2 (6.3–12.9)
Itching	6	5.6 (2.1–11.7)	3	2.7 (0.6–7.8)	4	3.7 (1.0–9.2)	13	4.0 (2.1–6.7)
Rash	7	6.5 (2.6–12.9)	1	0.9 (0.0–5.0)	0	0.0 (0.0–3.4)	8	2.5 (1.1–4.8)
Weakness	3	2.8 (0.6–7.9)	1	0.9 (0.0–5.0)	5	4.6 (1.5–10.5)	9	2.8 (1.3–5.2)
Cough	19	17.6 (10.9–26.1)	6	5.5 (2.0–11.5)	5	4.6 (1.5–10.5)	30	9.2 (6.3–12.9)
Vertigo	1	0.9 (0.0–5.1)	0	0.0 (0.0–3.3)	1	0.9 (0.0–5.1)	2	0.6 (0.1–2.2)
Dryness of mouth	6	5.6 (2.1–11.7)	1	0.9 (0.0– 5.0)	13	12.0 (6.6–19.7)	20	6.1 (3.8–9.3)
Unsolicited systemic AE^*^								
Post-any dose	29/112	25.9 (18.1–35.0)	21/112	18.8 (12.0–27.2)	23/112	20.5 (13.5–29.2)	73/336	21.7 (17.4–26.5)

CI, confidence interval; n, number of participants reporting the specified AE; M, number of participants with data available.

Solicited systemic reactions post any dose: 1–4 years, M = 108; 5–14 years, M = 110; ≥15 years, M = 108.

* Within 14 days post-dose 1 and within 30 days post-dose 2.

No immediate unsolicited systemic AEs were reported within 30 minutes following the first or second vaccine dose. The most frequently reported unsolicited AEs were infections and infestations in the 1–4 and 5–14 years age groups; these occurred in 6.3% and 11.6% of participants aged 1–4 years (mainly influenza and nasopharyngitis) post-dose 1 and post-dose 2, respectively, and 4.5% and 3.6% of participants aged 5–14 years (mainly varicella and nasopharyngitis) post-dose 1 and post-dose 2. In the ≥15 years age group, nervous system disorders (mainly headache) were the most frequently reported unsolicited AEs reported in 6.3% and 3.6% of participants post-dose 1 and post-dose 2, respectively. Most unsolicited AEs were of Grade 1 intensity, began after Day 4 and did not last more than 7 days. No unsolicited systemic AEs were assessed as related to vaccination, and none were serious AEs. Two unsolicited AEs were rated as Grade 3 intensity in the 5–14 years age group (headache and fever, both post-dose 2) and 2 in the ≥15 years age group (pain in right arm post-dose 1 and fever post-dose 2).

No AE led to discontinuation, and no serious adverse event or death was reported during the trial.

### Immunogenicity

**Figure 2.**
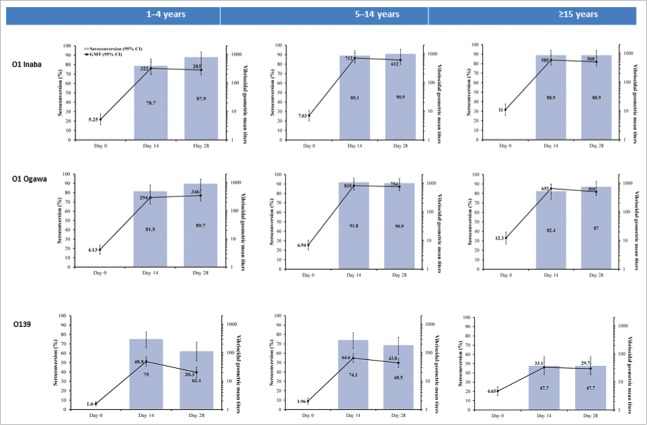
Vibriocidal antibody titers and proportion of participants with a ≥4-fold rise from baseline (seroconversion) (Full Analysis Set).

GMTs against *V. cholerae* serotypes (O1 Inaba, O1 Ogawa and O139) increased from baseline to Day 28 in all age groups (Table 3 and Fig. 2). GMTs (baseline and post-vaccination) against O139 were lower than those against O1 Inaba or Ogawa.

**Table 3.** Geometric mean titers, fold-change and seroconversion rates for *V. cholera* serogroups, by age group (Full Analysis Set).

	1–4 years (N = 112)			5–14 years (N = 112)		≥15 years (N = 112)	
	M or n/M	Mean titer, ratio or % (95% CI)		M or n/M	Mean titer, ratio or % (95% CI)	M or n/M	Mean titer, ratio or % (95% CI)
O1 Inaba							
GMT, 1/dil							
D0	112		5.25 (3.37–8.20)	112	7.03 (4.56–10.8)	112	11.0 (6.92–17.6)
D14	108		322 (197–528)	110	712 (483–1050)	108	585 (388–883)
D28	107		283 (191–419)	110	612 (426–880)	108	508 (365–708)
Individual ratio titers							
D14/ D0	108		63.2 (36.9–108)	110	111 (67.1–183)	108	55.6 (33.7–91.6)
D28/ D0	107		54.8 (35.1–85.4)	110	95.2 (61.0–149)	108	48.3 (30.3–76.9)
Seroconversion (≥ 4-fold rise)							
D14/D0	85/108		78.7 (69.8–86.0)	98/110	89.1 (81.7–94.2)	96/108	88.9 (81.4–94.1)
D28/D0	94/107		87.9 (80.1–93.4)	100/110	90.9 (83.9–95.6)	96/108	88.9 (81.4–94.1)
O1 Ogawa							
GMT (1/dil)							
D0	112		4.13 (2.85–5.97)	112	6.94 (4.66–10.3)	112	12.3 (7.59–19.8)
D14	108		294 (172–503)	110	818 (566–1183)	108	652 (453–940)
D28	107		346 (223–536)	110	754 (553–1028)	108	501 (370–679)
Individual ratio titers							
D14/D0	108		76.1 (44.5–130)	110	119 (75.2–187)	108	50.8 (31.3–82.4)
D28/D0	107		88.5 (54.0–145)	110	109 (71.4–167)	108	39.0 (24.5–62.2)
Seroconversion (≥ 4-fold rise)							
D14/D0	88/108		81.5 (72.9–88.3)	101/110	91.8 (85.0–96.2)	89/108	82.4 (73.9–89.1)
D28/D0	96/107		89.7 (82.3–94.8)	100/110	90.9 (83.9–95.6)	94/108	87.0 (79.2–92.7)
O139							
GMT (1/dil)							
D0	112		1.60 (1.33–1.91)	110	1.96 (1.53–2.50)	111	4.65 (3.20–6.76)
D14	108		48.8 (33.4–71.3)	110	64.6 (45.3–92.2)	108	33.1 (21.9–50.1)
D28	103		20.3 (13.5–30.6)	110	43.8 (30.1–63.7)	108	29.7 (19.9–44.4)
Individual ratio titers							
D14/ D0	108		30.3 (20.4–45.0)	108	32.4 (21.5–48.8)	107	7.23 (4.81–10.9)
D28/ D0	103		13.0 (8.65–19.6)	108	22.6 (15.0–34.1)	107	6.52 (4.40–9.64)
Seroconversion (≥ 4-fold rise)							
D14/D0	81/108		75.0 (65.7–82.8)	80/108	74.1 (64.8–82.0)	51/107	47.7 (37.9–57.5)
D28/D0	64/103		62.1 (52.0–71.5)	74/108	68.5 (58.9–77.1)	51/107	47.7 (37.9–57.5)

CI: confidence interval. M: number of participants with available data. n: number of participants with ≥4-fold rise in titers at the specified timepoint.

Marked increases in GMTs from baseline were observed post-dose 1 for O1 Inaba and Ogawa, with highest titers at day 14 in the 5–14 years group, compared with the 1–4 years group and the ≥15 years age group (Table 3 and Fig. 2). Post-dose 2, no further increases in GMTs from post-dose 1 levels were observed for any of the serotypes, with the exception of a slight increase in titers to O1 Ogawa in the 1–4 years age group (Table 3 and Fig. 2).

The percentage of participants with seroconversion at Days 14 and 28 (4-fold or greater increase in titers relative to baseline) was high for O1 Inaba and Ogawa for all age groups (Table 3 and Fig. 2). Post-dose 2 (Day 28), seroconversion rates for O1 Inaba ranged from 87.9% (1–4 years) to 90.9% (5–14 years) and for O1 Ogawa, from 87.0% (≥15 years) to 90.9% (5–14 years). Day 28 seroconversion rates were lower for O139 (ranging from 47.7% for ≥15 year-olds to 68.5% for 5–14 years) than the other serotypes.

Among participants with baseline titers ≤80, GMT ratio titers and seroconversion rates at Days 14 and 28, relative to baseline, tended to be higher than those observed for total participants for each age group (Supplementary Table S1). This observation was particularly marked for the older age group (≥15 years old).

In complementary analyses, total participants were reclassified based on the age groups 1–4, 5–17 and ≥18 years as used in a number of previous OCV studies.^7^^,^^10^ The observed trends were similar to those observed for the 1–4, 5–14 and ≥15 years age groups, with marked increases in GMTs from baseline to post-dose 1, the highest GMTs being for the 5–17 years age group. There was also a consistent trend towards similar or slightly lower GMTs post-dose 2 compared to post-dose 1 in the two older age groups (5–17 and ≥18 years), consistent with observations for the 5–14 and ≥15 years age groups. Seroconversion rates remained high across age groups for the O1 serotypes (Supplementary Table S2).

## Discussion

In this study, no safety concerns were identified with a 2-dose regimen of OCV in healthy participants in the Dominican Republic. The safety profile observed in our study is consistent with previous observations using the same dose and regimen in adults and children in Haiti,^7^ Ethiopia,^6^ India and Bangladesh.^4^^,^^11^

The OCV was immunogenic in all age groups in the current study, with robust increases in GMTs and high seroconversion rates. These findings support previous observations of robust vibriocidal antibody responses with OCV in neighbouring Haiti (on the other side of the Island of Hispanola), a country with only recent history of exposure to *V. cholerae*.^7^ In our study, baseline GMTs against all serotypes were low, but appeared to be of a similar magnitude to those against O1 Inaba and O1 Ogawa (O139 GMTs not reported) in the Haitian study. In addition, the baseline GMTs against all three serotypes appeared to increase with age in our study. There was a robust increase in GMTs after the first OCV dose, but no subsequent increases after the second dose, except for a limited non-significant increase against O1 Ogawa in the 1–4 years age group. In the Haitian study, vibriocidal GMTs increased further after the second dose, but not significantly; furthermore, no further boosting after the second dose was observed for O-specific polysaccharide serum IgA responses.^7^ However, vibriocidal antibody GMTs in the Haitian study were assessed seven days after each OCV dose as opposed to 14 days in the present study, which may in part explain these apparent differences.

Baseline titers have been shown to influence serotype-specific vibriocidal responses in both endemic^10^ and outbreak prone settings,^12^ whereby study participants with high baseline titers (>80) tended to have lower fold-increases than those with low titers (≤80) irrespective of age. Consistent with those previous observations, GMT ratio titers and seroconversion rates were particularly high among participants with baseline titers ≤80 in our study, compared to those for participants overall. Due to the small numbers of participants with baseline titers >80, we could not perform a meaningful comparison with this subgroup alone.

The lack of significant boost in immune response after the second dose relative to the first, and in some cases a decrease, has previously been observed in other studies of OCV undertaken in endemic and hyper-endemic countries.^6^^,^^12-14^ This was also observed when the interval between the 2 OCV doses was extended to 28 days.^11^ A possible explanation for these observations could be related to the higher antigenic lipopolysaccharide (LPS) content of reformulated OCVs, containing approximately twice the amount of LPS compared to the older generation oral whole-cell recombinant B subunit cholera vaccine,^15^ resulting in a stronger immune response following the first dose.^11^^,^^16^ It has been hypothesized that this first dose of the vaccine may stimulate an immune response in the intestinal mucosa and block uptake of the second dose, or block antibody production, and thus the observed levels may be due to the natural waning of antibodies.^11^^,^^13^ It is also possible that a booster-like effect may have occurred with the first dose due to previous exposure to cholera in an endemic setting.^11^ The significance of this lack of boost in the immune response after the second dose is unclear. Further insight on the duration of the immune response following single-dose and two-dose regimens would be needed to help clarify a potential impact on immunity over time.

Previous studies in hyper-endemic settings of India have reported much higher baseline GMTs and higher immune responses with OCV than in our study.^11^^,^^13^^,^^17^ This difference was particularly marked among baseline GMTs for adult age groups (≥18 years old) in the Indian studies compared to the older age group (≥15 years) in our study, with GMTs 13–29-fold higher for O1 serogroups and 41–70-fold higher for O139 in the Indian studies^11^^,^^13^^,^^17^ compared to findings in the current study, except for similar GMTs observed for O139 in one Indian study.^11^ These differences in baseline vibriocidal antibody GMTs are consistent with the higher exposure rate in India. After the first dose, vibriocidal antibody GMTs in adults were higher (about 2–3-fold for the O1 Inaba and O1 Ogawa serotypes and 8- and 9-fold for O139) in the Indian studies than in the current study,^11^^,^^13^^,^^17^; with the exception of lower O139 GMTs reported in one Indian study.^11^ Similarly higher-fold increases were observed in Indian children (1–17 years old) compared with children in the current study after the first dose of OCV, except for GMTs against O1 Inaba in one Indian study^17^ and GMTs against O139 reported in another Indian study,^11^ which were similar or lower than those reported in the current study, respectively.

Other countries with lower cholera endemicity than India have also reported, in general, higher baseline GMTs across the three serotypes than our study.^6^^,^^14^^,^^12^ In the Philippines, baseline GMTs for adults were 3–6-fold higher for O1 Inaba and O1 Ogawa than in our study, but with similar O139 titers^14^; baseline GMTs were also higher in Filipino children for the three serotypes (about 2-fold higher) than our study. However, vibriocidal antibody GMTs after the first dose in Filipino adults were ≤2-fold higher for O1 Inaba and O1 Ogawa than in our study and lower for O139; and post-dose 1 GMTs in Filipino children were similar to those in our study. In South Sudan, baseline GMTs across similar age groups as in our study were 2–4-fold higher for the O1 Inaba and O1 Ogawa serotypes than in our study (O139 data not reported).^12^ In South Sudan's neighbour, Ethiopia, baseline GMTs in adults were also higher for O1 Inaba and O1 Ogawa than in our study (similar O139 titers), and in children (1–17 years) were similar to those observed in the current study, for all serotypes.^6^ In both South Sudan and Ethiopia, however, vibriocidal antibody GMTs after the first dose were lower than in our study for all serotypes in both adults and children.^6^^,^^12^

While baseline vibriocidal antibody GMTs would be expected to differ between countries as a result of varying levels of continual background exposure to cholera, comparison between studies should be made with caution because of the variability in the vibriocidal assay across laboratories. The vibriocidal antibody assay is the most widely used and useful surrogate marker of intestinal immune response after oral vaccine administration and considered the ‘gold standard’ measure. Although the intestinal secretory IgA is a better predictor of protection, it is not practical for use in a large clinical trial.^18^^,^^19^

The reasons for lower GMTs against O139 relative to O1 Inaba and O1 Ogawa serotypes in our study and other OCV studies are unclear, but may reflect poorer responses to the O139 vaccine antigen or a lower sensitivity of the vibriocidal antibody assay, which may be affected by the presence of a capsule in the O139 strain.^20^^,^^21^ In addition, the relative differences in baseline GMTs between the serotypes could be expected to be indicative of the relative differences in the circulation of the different serotypes. In neighbouring Haiti, sentinel surveillance of 10 public hospitals located in each of the country's administrative departments between 1 November 2011 and 30 October 2012 showed that *V. cholerae* serogroup O1 caused 45.9% (210/458) of acute watery diarrhea, with the O1 Ogawa serotype responsible for 98.6% of the cholera isolates (207/210) and O1 Inaba for the remaining 1.4% (3/210). The lack of O139 detection in the Haitian sentinel surveillance study would be consistent with the very low circulation of this serotype on the Island of Hispanola, and the very low baseline GMTs observed for O139 in our study, particularly in children.

In conclusion, the current study shows an acceptable safety profile and robust immunogenicity of OCV in all age groups in the Dominican Republic. These findings support previous observations of OCV safety and immunogenicity in both epidemic and historically endemic settings.

## Methods

### Study design and participants

This was a phase III, open-label, descriptive study undertaken at two centres in the Dominican Republic between 27 April 2015 and 16 September 2015. This study is registered on www.clinicaltrials.gov (NCT02434822). The study was conducted in accordance with the Declaration of Helsinki and International Conference on the Harmonization-Good Clinical Practice. The study was approved by el Consejo Nacional de Bioetica En Salud (CONABIOS) and the Fundacion Dominicana de Infectologia. Informed consent forms were signed by the parent(s) or legal guardians for participants aged 1–17 years, and those aged ≥18 years signed them independently. Participants aged 9–17 years also signed the assent form.

Healthy individuals aged ≥1 year were eligible for inclusion. Toddlers aged ≤2 years had to have been born at full term (≥37 weeks’ gestation) and/or with a birth weight ≥2.5 kg. Women of child-bearing potential were required to use an effective method of contraception, or be sexually abstinent for at least 4 weeks pre- and post- vaccination. Exclusion criteria included: pregnancy; receipt of blood or blood products in the past three months; receipt in the last five years of cholera vaccination with the trial vaccine or another vaccine; receipt of any vaccine within four weeks preceding or following trial vaccination, except for influenza vaccination which could be received up to two weeks before or after trial vaccination; known hypersensitivity to any vaccine component; suspected or confirmed immunosuppressive or immunodeficient condition; acute febrile illness on the day of vaccination (body temperature ≥38°C) or diarrhea within six weeks prior to enrollment.

### Vaccines and vaccine administration

Participants received two doses of open-label OCV vaccine (Shanchol™) 14 days apart (Days 0 and 14 [+2 days]). Each dose of OCV was administered as a 1.5 mL buffered solution containing thiomersal 0.02% (w/v) and 2100 ELISA units (EU) of lipopolysaccharide (LPS) from five strains of formaldehyde- or heat-killed *V.cholerae*, as follows: O1 Inaba el Tor strain Phil 6973 (600 EU LPS); O1 Ogawa classical strain Cairo 50 (300 EU LPS); O1 Inaba classical strain Cairo 48 (300 EU LPS); O139 strain 4260B (600 EU LPS).

The vaccine was presented as a white suspension, which was poured into the recipients’ mouth, followed by a drink of water if needed.

### Assessment of safety and reactogenicity

All participants were observed for 30 minutes following vaccination on Days 0 and 14 to monitor for any immediate unsolicited systemic AEs. Participants or their parent(s) or legal guardians were provided with diary cards and a thermometer to record any solicited systemic reactions occurring up to 7 days after each vaccination and to grade their severity: Grade 1 (body temperature 38.0–38.4°C) to 3 (≥39°C) for fever; Grade 1 (mild) to 4 (emergency room visit or hospitalization) for nausea, vomiting, diarrhea, abdominal pain, itching, rash, weakness, cough, vertigo and dryness of mouth.

Unsolicited AEs were recorded in the diary cards up to 14 days (+2 days) after the first vaccination and 30 days (+7 days) after the second vaccination. Severity was graded on a 3-point scale as follows: Grade 1, no interference with activity; Grade 2, some interference with activity; and Grade 3, prevention of daily activity. Unsolicited AEs were assessed as either related or not related to vaccination by the Investigator. Serious AEs were monitored throughout the trial, and were assessed as related or not related to vaccination by the study Investigator.

### Immunogenicity assessment

Blood samples (3 mL) were drawn before (pre-vaccination) and 14 days (+2 days) after each vaccine dose. Samples were stored at room temperature for two hours and centrifuged to obtain sera. Sera were shipped frozen using dry ice to maintain frozen state. Serum vibriocidal antibodies to strains of *V. cholerae* (O1 El Tor Inaba, O1 El Tor Ogawa and O139) were measured prior to the first vaccination (Day 0), at Day 14 and Day 28 (primary immunogenicity endpoints).

A Guinea-pig complement serum (C300–500; Rockland™ Gilbertsville, PA, USA; Lot No. 32066) mediated vibriocidal antibody assay using the microtiter technique was performed for antibody detection at a central laboratory, the International Vaccine Institute (IVI) in Seoul, Korea. Two-fold serial dilutions of pre- and post-vaccination samples were analysed side-by-side in duplicates. The final titer was taken as the mean of the duplicate measurements. Titers were adjusted in relation to a reference serum specimen included in each test to compensate for variations between analyses on different occasions. The assay was repeated if a ≥2-fold difference was noted between the results of the duplicate tests. Seroconversion was defined as a 4-fold or greater increase in titers at Days 14 or 28 relative to baseline.

### Statistical Methods

This study was descriptive without hypothesis testing. Analyses were stratified by age (1–4, 5–14 and ≥15 years). In complementary analyses, data were additionally stratified by age groups 1–4, 5–17 and ≥18 years. The total number of participants enrolled was determined arbitrarily.

Safety following any dose was assessed on the Safety Analysis Set, defined as all participants who received at least one dose of the vaccine; safety post-dose 1 or 2 was assessed for participants who received the corresponding dose. Immunogenicity was assessed on the Full Analysis Set, which included all participants who received at least one dose and in practice was identical to the Safety Analysis Set for any dose.

GMTs and 95% confidence intervals (CI) values were calculated on Log_10_ scale for normal distribution (using Student's *t* distribution with n-1 degree of freedom). CIs for seroconversion rates were calculated using the Clopper-Pearson method.^22^ All statistical analyses were performed using the SAS® software, Version 9.2 (SAS Institute, Cary, North Carolina, USA).

## Supplementary Material

KHVI_A_1430540_Supplemental.docx
