# Supplementary material for: Bivalent oral cholera vaccine in participants aged 1 year and older in the Dominican Republic: A phase III, single-arm, safety and immunogenicity trial
Source: Hum Vaccin Immunother. 2018 Feb 22;14(6):1403–11. doi: 10.1080/21645515.2018.1430540 (PMC6037475; doi:10.1080/21645515.2018.1430540)
Supplement: KHVI_A_1430540_Supplemental.docx [file khvi-14-06-1430540-s001.docx]

**Supplementary Tables.**

**Supplementary Table S1.** Geometric mean titers, individual ratio titers and seronversion rates for *V. cholera* serogroups, by age group (participants with baseline titers ≤80).

|  |  | **1 to 4 years (N=112)** | | | **5 to 14 years (N=112)** | | **≥15 years (N=112)** | |
| --- | --- | --- | --- | --- | --- | --- | --- | --- |
|  |  | **M or n/M** | **Mean titer, ratio or % (95% CI)** | | **M or n/M** | **Mean titer, ratio or % (95% CI)** | **M or n/M** | **Mean titer, ratio or % (95% CI)** |
| **O1 Inaba** | | | | | | | | |
| **GMT, 1/dil** | |  |  | |  |  |  |  |
|  | **D0** | 94 | 2.19 (1.68–2.85) | | 95 | 3.35 (2.42–4.63) | 85 | 3.55 (2.51–5.01) |
|  | **D14** | 91 | 229 (133–395) | | 95 | 604 (394–925) | 82 | 405 (254–648) |
|  | **D28** | 90 | 203 (137–300) | | 95 | 492 (337–720) | 82 | 385 (270–549) |
| **Individual ratio titers** | |  |  | |  |  |  |  |
|  | **D14/ D0** | 91 | 107 (61.3–188) | | 95 | 180 (110–297) | 82 | 122 (72.8–203) |
|  | **D28/ D0** | 90 | 94.8 (63.2–142) | | 95 | 147 (95.3–227) | 82 | 116 (75.4–177) |
| **Seroconversion (≥ 4-fold rise)** | |  |  | |  |  |  |  |
|  | **D14/D0** | 74/91 | 81.3 (71.8–88.7) | | 90/95 | 94.7 (88.1–98.3) | 77/82 | 93.9 (86.3–98.0) |
|  | **D28/D0** | 84/90 | 93.3 (86.1–97.5) | | 91/95 | 95.8 (89.6–98.8) | 81/82 | 98.8 (93.4–100.0) |
| **O1 Ogawa** | | | | | | | | |
| **GMT (1/dil)** | |  | |  |  |  |  |  |
|  | **D0** | 102 | | 2.68 (2.01–3.55) | 97 | 3.92 (2.82–5.46) | 89 | 4.52 (3.16–6.46) |
|  | **D14** | 100 | | 253 (144–443) | 95 | 751 (498–1133) | 85 | 509 (333–778) |
|  | **D28** | 99 | | 322 (206–503) | 95 | 678 (484–951) | 85 | 402 (284–568) |
| **Individual ratio titers** | |  | |  |  |  |  |  |
|  | **D14/ D0** | 100 | | 93.1 (53.4–162) | 95 | 195 (126–303) | 85 | 111 (70.3–177) |
|  | **D28/ D0** | 99 | | 118 (73.4–189) | 95 | 176 (118–263) | 85 | 88.0 (58.0–133) |
| **Seroconversion (≥ 4-fold rise)** | |  | |  |  |  |  |  |
|  | **D14/D0** | 83/100 | | 83.0 (74.2–89.8) | 91/95 | 95.8 (89.6–98.8) | 81/85 | 95.3 (88.4–98.7) |
|  | **D28/D0** | 90/99 | | 90.9 (83.4–95.8) | 92/95 | 96.8 (91.0–99.3) | 84/85 | 98.8 (93.6–100.0) |
| **O139** | | | | | | | | |
| **GMT (1/dil)** | |  | |  |  |  |  |  |
|  | **D0** | 110 | | 1.48 (1.28–1.71) | 106 | 1.66 (1.37–2.02) | 94 | 2.44 (1.84–3.23) |
|  | **D14** | 106 | | 47.7 (32.4–70.0) | 106 | 61.9 (42.9–89.2) | 92 | 24.0 (15.3–37.8) |
|  | **D28** | 102 | | 19.8 (13.1–29.9) | 106 | 41.1 (28.0–60.2) | 92 | 21.6 (13.9–33.4) |
| **Individual ratio titers** | |  | |  |  |  |  |  |
|  | **D14/ D0** | 106 | | 32.1 (21.6–47.6) | 104 | 36.5 (24.3–54.9) | 91 | 9.84 (6.28–15.4) |
|  | **D28/ D0** | 102 | | 13.3 (8.79–20.0) | 104 | 25.0 (16.5–37.8) | 91 | 8.88 (5.78–13.6) |
| **Seroconversion (≥ 4-fold rise)** | |  | |  |  |  |  |  |
|  | **D14/D0** | 81/106 | | 76.4 (67.2–84.1) | 80/104 | 76.9 (67.6–84.6) | 51/91 | 56.0 (45.2–66.4) |
|  | **D28/D0** | 64/102 | | 62.7 (52.6–72.1) | 74/104 | 71.2 (61.4–79.6) | 51/91 | 56.0 (45.2–66.4) |

CI: confidence interval. M: number of participants with available data. n: number of participants with ≥4-fold rise in titers at the specified timepoint.

**Supplementary Table S2.** Geometric mean titers, individual ratio titers and seronversion rates for *V. cholera* serogroups, by age groups 1–4, 5–17 and ≥18 years (Full Analysis Set).

|  |  | **1 to 4 years (N=112)** | | | **5 to 17 years (N=117)** | | | **≥18 years (N=107)** | | |
| --- | --- | --- | --- | --- | --- | --- | --- | --- | --- | --- |
|  |  | M or n/M | Mean titer, ratio or % (95% CI) | | M or n/M | | Mean titer, ratio or % (95% CI) | M or n/M | | Mean titer, ratio or % (95% CI) |
| **O1 Inaba** | | | | | | | | | | |
| **GMT (1/dil)** | |  | |  |  | |  |  | |  |
|  | **D0** | 112 | | 5.25 (3.37–8.20) | 116 | | 6.78 (4.46–10.3) | 108 | | 11.7 (7.22–18.8) |
|  | **D14** | 108 | | 322 (197–528) | 113 | | 676 (456–1003) | 105 | | 615 (410–924) |
|  | **D28** | 107 | | 283 (191–419) | 113 | | 595 (416–851) | 105 | | 522 (373–729) |
| **Individual ratio titers** | |  | |  |  | |  |  | |  |
|  | **D14/ D0** | 108 | | 63.2 (36.9–108) | 113 | | 107 (65.2–176) | 105 | | 56.5 (34.1–93.6) |
|  | **D28/ D0** | 107 | | 54.8 (35.1–85.4) | 113 | | 94.2 (60.9–146) | 105 | | 47.9 (29.7–77.1) |
| **Seroconversion (≥ 4-fold rise)** | |  | |  |  | |  |  | |  |
|  | **D14/ D0** | 85/108 | | 78.7 (69.8–86.0) | 100/113 | | 88.5 (81.1–93.7) | 94/105 | | 89.5 (82.0–94.7) |
|  | **D28/ D0** | 94/107 | | 87.9 (80.1–93.4) | 103/113 | | 91.2 (84.3–95.7) | 93/105 | | 88.6 (80.9–94.0) |
| **O1 Ogawa** | | | | | | | | | | |
| **GMT (1/dil)** | |  | |  |  |  | |  |  | |
|  | **D0** | 112 | | 4.13 (2.85–5.97) | 116 | 6.78 (4.59–10.0) | | 108 | 12.8 (7.86–21.0) | |
|  | **D14** | 108 | | 294 (172–503) | 113 | 803 (559–1153) | | 105 | 661 (456–960) | |
|  | **D28** | 107 | | 346 (223–536) | 113 | 732 (539–995) | | 105 | 511 (375–697) | |
| **Individual ratio titers** | |  | |  |  |  | |  |  | |
|  | **D14/ D0** | 108 | | 76.1 (44.5–130) | 113 | 117 (75.3–183) | | 105 | 50.1 (30.5–82.4) | |
|  | **D28/ D0** | 107 | | 88.5 (54.0–145) | 113 | 107 (70.7–162) | | 105 | 38.8 (24.0–62.5) | |
| **Seroconversion (≥ 4-fold rise)** | |  | |  |  |  | |  |  | |
|  | **D14/ D0** | 88/108 | | 81.5 (72.9–88.3) | 104/113 | 92.0 (85.4–96.3) | | 86/105 | 81.9 (73.2–88.7) | |
|  | **D28/ D0** | 96/107 | | 89.7 (82.3–94.8) | 103/113 | 91.2 (84.3–95.7) | | 91/105 | 86.7 (78.6–92.5) | |
| **O139** | | | | | | | | | | |
| **GMT (1/dil)** | |  | |  |  |  | |  |  | |
|  | **D0** | 112 | | 1.60 (1.33–1.91) | 114 | 1.93 (1.52–2.44) | | 107 | 4.88 (3.32–7.18) | |
|  | **D14** | 108 | | 48.8 (33.4–71.3) | 113 | 62.7 (44.1–89.2) | | 105 | 33.6 (22.1–51.1) | |
|  | **D28** | 103 | | 20.3 (13.5–30.6) | 113 | 42.4 (29.3–61.4) | | 105 | 30.4 (20.2–45.8) | |
| **Individual ratio titers** | |  | |  |  |  | |  |  | |
|  | **D14/ D0** | 108 | | 30.3 (20.4–45.0) | 111 | 31.8 (21.2–47.6) | | 104 | 7.06 (4.67–10.7) | |
|  | **D28/ D0** | 103 | | 13.0 (8.65–19.6) | 111 | 22.1 (14.8–33.2) | | 104 | 6.43 (4.31–9.58) | |
| **Seroconversion (≥ 4-fold rise)** | |  | |  |  |  | |  |  | |
|  | **D14/ D0** | 81/108 | | 75.0 (65.7–82.8) | 82/111 | 73.9 (64.7–81.8) | | 49/104 | 47.1 (37.2–57.2) | |
|  | **D28/ D0** | 64/103 | | 62.1 (52.0–71.5) | 76/111 | 68.5 (59.0–77.0) | | 49/104 | 47.1 (37.2–57.2) | |

CI: confidence interval. M: number of participants with available data. n: number of participants with ≥4-fold rise in titers at the specified timepoint.
